# Bibliometric analysis of the Vogt‒Koyanagi‒Harada disease literature

**DOI:** 10.1007/s10792-023-02815-x

**Published:** 2023-08-08

**Authors:** Liangpin Li, Liyun Yuan, Xueyan Zhou, Xia Hua, Xiaoyong Yuan

**Affiliations:** 1 Clinical College of Ophthalmology, Tianjin Medical University, Tianjin, 300020 China; 2 Tianjin Key Laboratory of Ophthalmology and Visual Science, Tianjin Eye Institute, Tianjin Eye Hospital, Tianjin, 300020 China; 3 School of Medicine, Nankai University, Tianjin, 300071 China; 4 Tianjin Aier Eye Hospital, Tianjin University, Tianjin, 300190 China

**Keywords:** Vogt‒Koyanagi‒Harada disease, Bibliometric, Inflammation, H-index

## Abstract

**Purpose:**

As an autoimmune disease, Vogt‒Koyanagi‒Harada disease (VKHD) is a main type of uveitis in many countries and regions, significantly impacting patient vision. At present, information regarding VKHD is still limited, and further research is needed. We conducted a bibliometric analysis to characterize the overall status, current trends, and current focus of VKHD research.

**Method:**

Literature published from 1975 to 2022 was obtained from the Web of Science core collection and analysed with the R-language packages Bibliometrix, VOSviewer, and CiteSpace software.

**Results:**

A total of 1050 papers on VKHD were retrieved from 261 journals, and 16,084 references were obtained from the papers in the original search. The average annual number of published articles was approximately 21.9, and the number of publications rapidly increased after 2004. The journal *Ocular Immunology and Inflammation* published the most papers on VKHD, while the *American Journal of Ophthalmology* has the highest citation frequency. The leading countries were Japan, China (PRC), and the United States of America (USA). Yang PZ from Chongqing Medical University was the most prolific and cited author. The most frequently cited study discussed revision of VKHD diagnostic criteria. An analysis of the highest frequency keywords showed that most research focused on the treatment, diagnosis, and pathogenesis of VKHD and its relationship with other related diseases. At present, the most urgent research direction is in the relationship between COVID-19 or COVID-19 vaccines and VKHD and the corresponding mechanisms underlying it.

**Conclusion:**

Utilizing dynamic and visualization tools, bibliometrics provides a clear depiction of the research history, development trends, and research hotspots in VKHD It serves as a valuable tool for identifying research gaps and areas that necessitate further exploration. Our study revealed potential directions for future VKHD research, including investigating specific molecular mechanisms underlying the disease, exploring the clinical utility of optical coherence tomography angiography and other diagnostic techniques, and conducting clinical research on novel therapeutic drugs.

**Supplementary Information:**

The online version contains supplementary material available at 10.1007/s10792-023-02815-x.

## Introduction

Vogt‒Koyanagi‒Harada disease (VKHD) is an autoimmune, multisystem inflammatory disease characterized by bilateral diffuse granulomatous uveitis involving the central nervous system, vestibular organs, and skin [1]. Swiss physician Alfred Vogt first reported VKHD in 1906, as well as Japanese researchers Harada and Koyanagi. To commemorate the contribution of these three physicians, the disease was jointly named after them. Other names include idiopathic uvea encephalitis and uveomeningoencephalitis, but they are rarely used. It is one of the most common types of uveitis among Asians, Native Americans, Hispanics, and Middle Easterners [2–6]. The course of the disease can be divided into four stages: prodromal, acute uveitis, chronic convalescent, and chronic recurrent stages, with chronic recurrence signifying permanently impaired visual acuity and poor prognosis [7]. The main therapy for patients with VKHD typically involves a combination of corticosteroids and immunosuppressive agents [8, 9]. Receiving proper treatment in the early stages of the disease can lead to favourable therapeutic outcomes [10]. Unfortunately, even now, many patients enter the chronic recurrent stage because they are not diagnosed in the early stages and, therefore, are not receiving the correct treatment. The immune response pathway follows a course characterized by T-cell-mediated autoimmunity, specifically targeting melanocyte or melanocyte-associated antigens. This immune reaction leads to inflammation within the choroidal layer [11]. There are many hypotheses for the aetiology of the disease, including involvement of genetic factors, virus infection triggers, the gut microbiome and cell-mediated immune mechanisms, but the exact aetiology and pathogenesis of VKHD are still unclear [10, 12].

Using mathematical and statistical methods, bibliometrics consists of quantitative and visual analyses of a specific topic based on published works [13]. The results of the analysis can be visualized in the form of statistical tables or graphs, which can help quickly reveal the hotspots and trends in a particular field, author cooperation networks, institutional cooperation networks, article correlation analysis, etc. Bibliometric analysis is widely used in the field of ophthalmology and provides significant assistance in both research and clinical settings. It has been applied to various areas, such as the study of anti-cataract medications, research on dry eye syndrome, and investigations into the development of myopia [14–16]. VKHD is a complex and multifaceted condition whose study involves a variety of topics, such as epidemiology, clinical manifestations, pathogenesis, diagnosis, and treatment. Bibliometric analysis allows a comprehensive evaluation of the literature on VKHD by analysing a large volume of publications. This analysis provides insights into the overall research landscape, trends, and knowledge gaps in the field. In this study, we aimed to analyse VKHD-related literature using the Web of Science (WoS) database and report a bibliometric analysis of the literature on VKHD to help researchers who are interested in quickly understanding the historical developments in the field, its research trends, and the most relevant journals.

## Methods

The search for papers included in this study was carried out in October 2022 through the WoS Core Collection provided by Thomson Reuters (Philadelphia, PA, USA). Only “articles” and” reviews” were included as document types. The kind of literature was limited to “English-language” papers. The search period was from January 1, 1975, to October 28, 2022. Data were downloaded from the WoS in “plain text” format with a “full record and cited references”. In addition, we excluded 1 book chapter, 25 online published papers, 25 conference transcripts, and 1 withdrawn publication. The final search formula was (((TS = (Vogt‒Koyanagi‒Harada)) OR (TS = (VKH disease)) OR (TS = (idiopathic uvea encephalitis)) OR (TS = (Uveomeningoencephalitis)) OR (AB = (Vogt‒Koyanagi‒Harada)) OR (AB = (VKH disease)) OR (AB = (idiopathic uvea encephalitis)) OR (AB = (Uveomeningoencephalitis)) AND (LA = (English)) AND (DT = (Article OR Review))). Within the WoS retrieval system, the “article” category encompasses research papers, concise communications, technical descriptions, chronicles, complete papers, and case reports (presented in the form of complete papers), which are published in academic journals and/or presented at symposiums or conferences. Furthermore, the document type “review” includes Reviews, Review of Literature, Mini-reviews, and Systematic reviews.

After thoroughly reading the titles, abstracts, and keywords of articles retrieved from the search, we eliminated studies with poor relevance to the research topic, that did not meet the inclusion criteria or met the exclusion criteria, where researchers contacted the preliminary screening literature, and that deviated from the txt format "fully recorded and cited references”. Each document included the basic information of the content, author, publication, and all citation information (where total citations refers to the overall frequency of citations that a document has received in the WoS database, while local citations indicate the total frequency of citations within the scope of the current retrieval. Since local citations can better highlight the value of a document within a specific research field, this study primarily focuses on local citations for reference.). The text format file was imported into CiteSpace (software version 5.7. R5W http://cluster.ischool.drexel.edu/~cchen/citespace/download) was used to filter duplicate studies and those that lack time information, and the final literature for research and analysis was obtained. The collected data were visualized using VOSviewer (Software, version 1.6.18) and R (Programming language, version 4.2.2). Then, data analysis and visualization were carried out using various programmes, including VOSviewer, CiteSpace, and the bibliometrix R package (available at http://www.bibliometrix.org with the code “ > library(bibliometrix) →  > biblioshiny”). Scimago Graphica (version 1.0.30) was selected to depict the world map.

Since the data in this study were derived from publicly available data in public databases (WoS) and did not involve new human or animal experiments, ethical proof was not needed.

## Result

### Distribution of articles by publication year

**Fig. 1:**
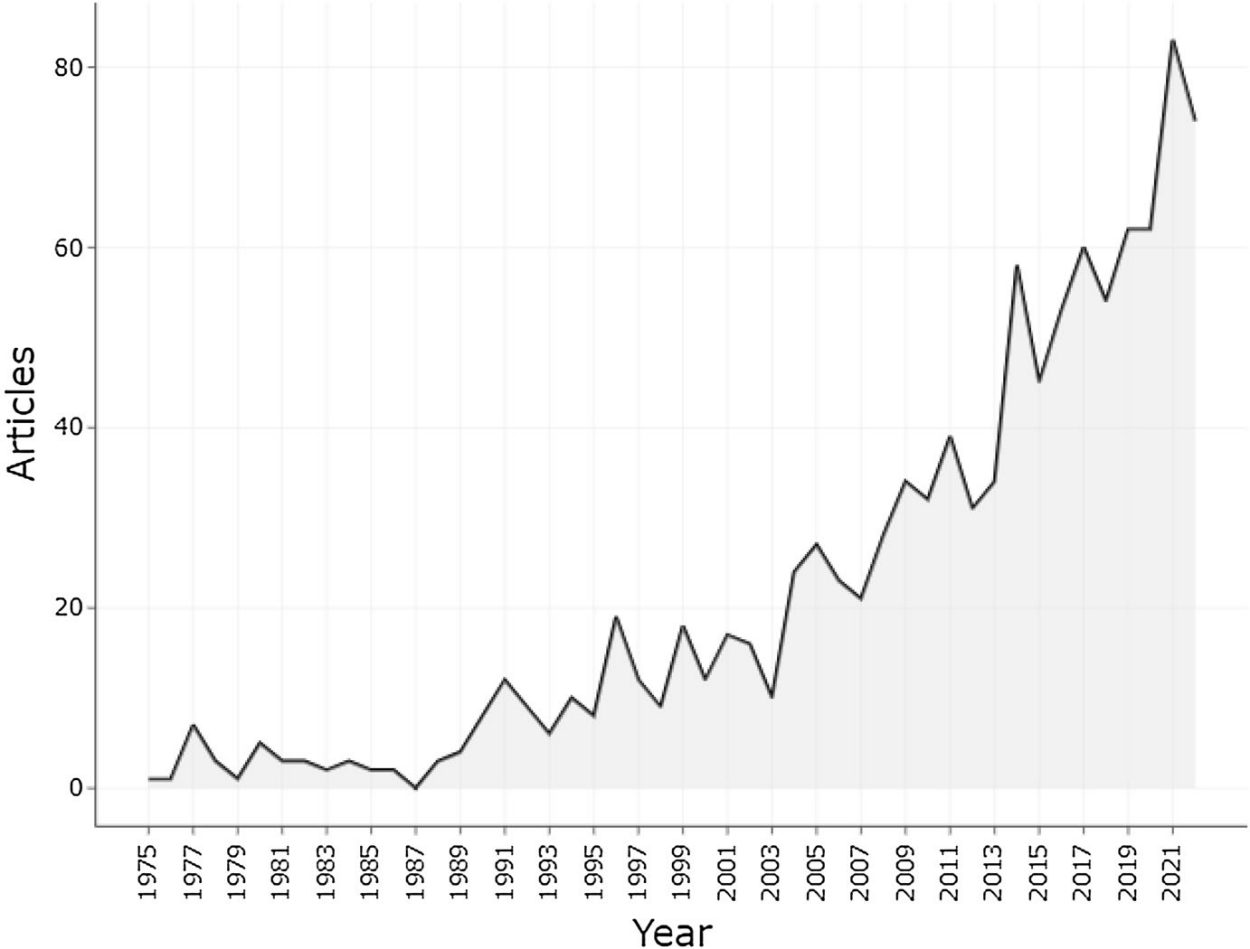
Number of publications per year

This study conducted a bibliometric analysis of 1050 VKHD-related literature searched from the WoS database, verified by CiteSpace software (1050 unique records, 0 duplicated records and 0 invalid records). The annual publication volume is shown in Fig. 1. Among them were 966 articles (92%) and 84 reviews (8%). The annual growth rate was 9.59%, and an average of 11 articles were published each year (Fig. 1). Before 1989, few studies on VKHD had been published annually. In addition, from 1989 to 2003, the number of papers related to the VKHD globally showed a relatively stable trend. Nevertheless, since 2004, the annual publication volume of VKHD has begun to exceed 20 and has increased thereafter. The number of studies shown for 2022 is slightly lower because the data still needed to be collected as of the time of analysis.

### Countries of origins

**Fig. 2:**
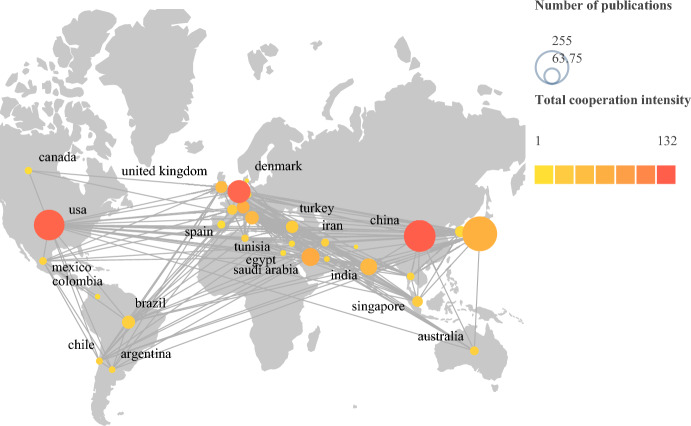
Map of international exchanges and cooperation. The size of the circles represents the number of posts, the lines between the circles represent mutual cooperation, and the darker the circle is, the more international communications and collaborations it has

Based on the R-language package bibliometrix, through a statistical analysis of the countries/regions included in the literature, we found that a total of 68 countries/regions have published literature on VKHD worldwide (based on the nationality of the corresponding author), and papers from the top 10 countries/regions had been cited frequently. Japan had the greatest number of articles (241 papers, 22.95%), with 5402 citations in total, while China ranked second (206 papers, 19.61%), with 3675 citations, and in third place was the United States (128 papers, 12.19%), with 3608 citations. These three countries accounted for 45.23% of the total. Countries with many published papers were also closely linked (Fig. 2; Table 1).

### Author distribution

According to the search results, 3455 authors participated in VKHD studies, of whom the 10 most prolific are shown in Table 2. Yang P.Z. from China published 109 papers, KIJLSTRA A. from the Netherlands published 101 papers, HOU S.P. from China published 45 papers, OHNO S. from Japan published 40 papers, and RAO N.A. from the United States published 30 papers. In addition, the citation frequency of RAO N.A. was the highest (1418), and Yang P.Z., KIJLSTRA A., and OHNO S. were also cited more than 1000 times. In the top 50 cited articles (Supplementary Table 1), it is noteworthy that RAO N.A. appears as the corresponding author in a total of 6 articles, followed by Yang P.Z. (5 articles) and Tamaki K. (2 articles).

**Table 1:** Top 10 counties or regions

Rank	Country or region	Counts	Citations
1	Japan	241	5402
2	China	206	3675
3	USA	128	3608
4	Saudi Arabia	58	1124
5	India	41	534
6	Brazil	32	817
7	Italy	27	432
8	Korea	23	262
9	Switzerland	23	442
10	Turkey	22	131

**Table 2:** Top 10 authors

Rank	Authors	H-index	Counts	Local cited
1	Yang PZ	26	109	1183
2	Kijlstra A	25	101	1094
3	Rao NA	21	30	1418
4	Ohno S	20	40	1019
5	Hou SP	18	45	364
6	Mochizuki M	17	24	402
7	Du LP	15	29	360
8	Abu El-Asrar AM	14	28	289
9	Li FZ	14	16	170
10	Chee SP	13	18	324

**Fig. 3:**
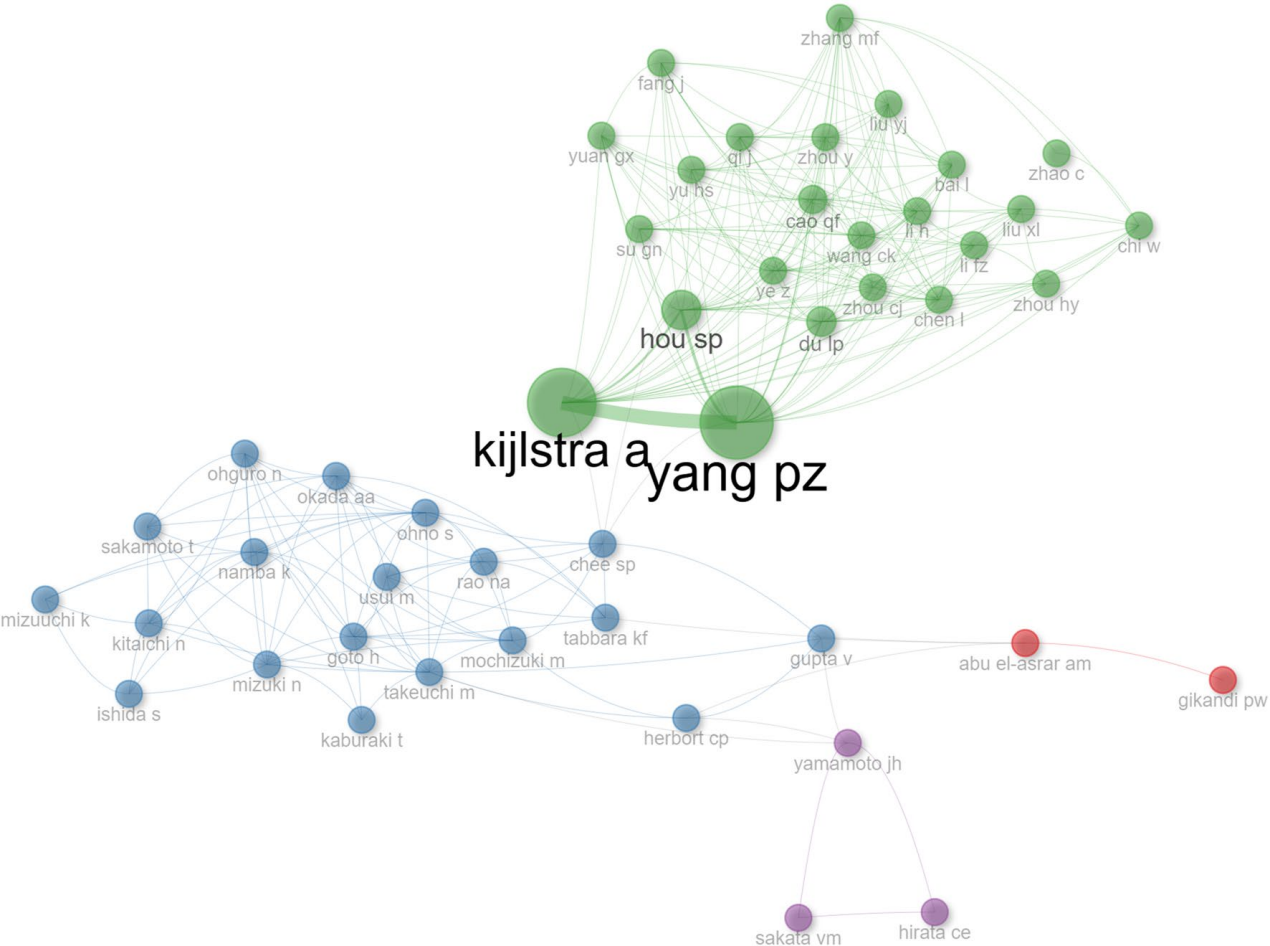
Connection diagram between the authors. The size of the dots represents the number of papers contributed, while the thickness of the lines represents the degree of connection

The H-index, also known as the H-factor, is a new method for evaluating academic achievement, wherein H stands for high citations. According to the H-index ranks of the authors, we find that the top five were Yang P.Z., Kijlstra A., Rao N.A., Ohno S., and Hou S.P. (Table 2). There was a close cooperative relationship among Yang P.Z., Kijlstra A. and Hou S.P. (Fig. 3, generated by Bibliometrix- Social structure), and cooperation between authors in this field is widespread.

Similar to the H-index, the impact factor (IF) is another important indicator. Supplementary Table 2 includes all the articles with an IF greater than 10. The highest IF in the list is attributed to an article published by Hou, S.P. et al. (with Yang P.Z. as the corresponding author) in NATURE GENETICS (IF = 41.307). In addition, as a corresponding author, KIJLSTRA A. has had three publications with an impact factor higher than 10.

### Institutions

**Fig. 4:**
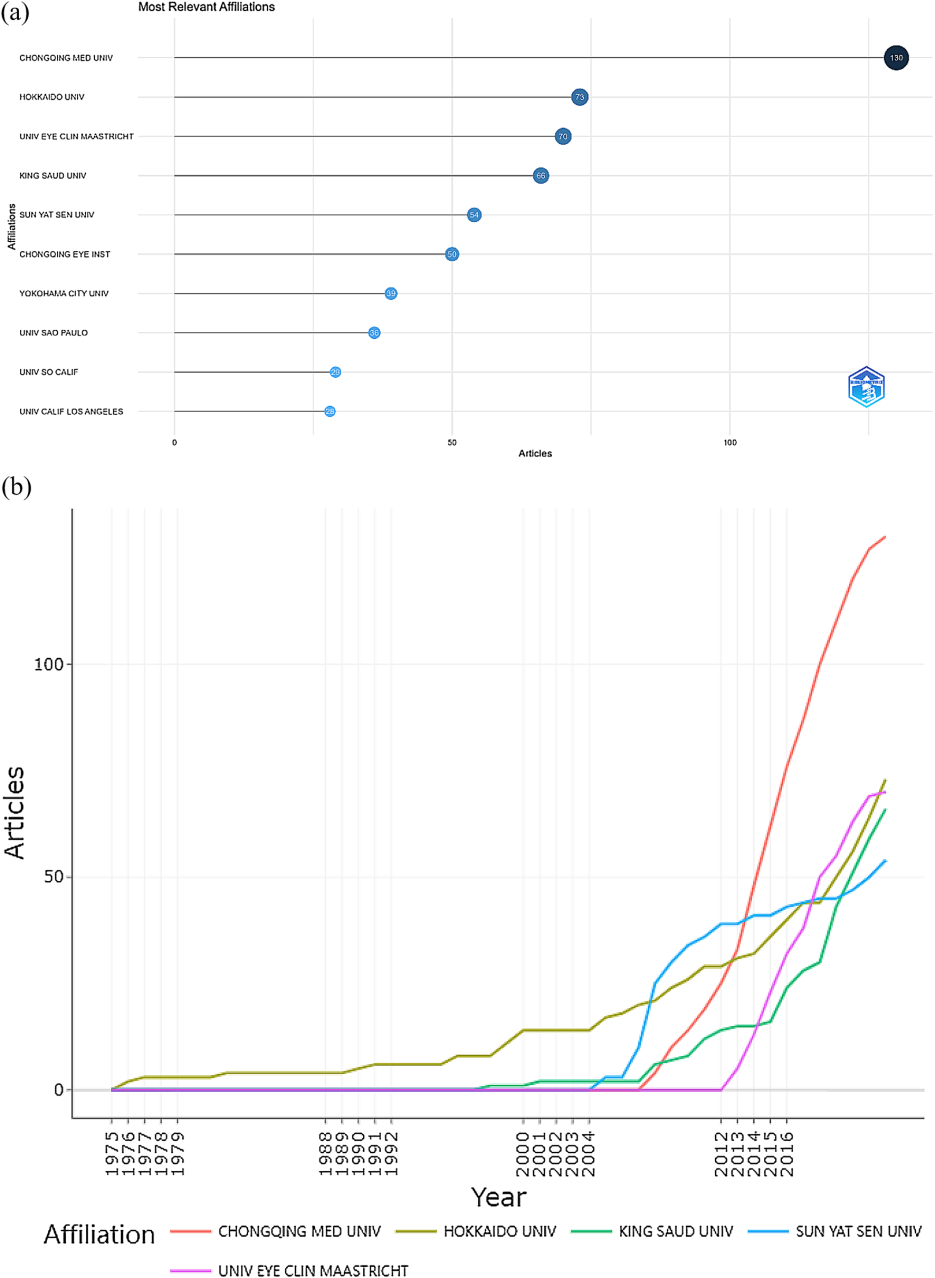
**a** Top 10 most prolific institutions; **b** number of articles published by institutions each year

Figure 4 shows the ranking of research institutions by the number of articles published, of which we list the top ten. This information is derived from the data analysed by the R package. According to the ranking of the number of papers issued by institution (Fig. 4a), Chongqing Medical University, where Yang P.Z. is located, ranks first, with a total of 130 papers, followed by Hokkaido University and University Eye Clinic Maastricht, with 73 and 70 papers, respectively. Since 2005, the number of papers published by Chongqing Medical University has increased dramatically. After 2014, it surpassed other institutions to become the institution with the largest annual average volume of papers (Fig. 4b). More detailed information can be found in Table 3.

**Table 3:** Top 10 most prolific organizations

Rank	Institutions	Counts	Citations
1	Chongqing Med Univ	130	5402
2	Hokkaido Univ	73	3675
3	Univ Eye Clin Maastricht	70	3608
4	King Saud Univ	66	1124
5	Sun Yat Sen Univ	54	906
6	Chongqing Eye Inst	50	534
7	Yokohama City Univ	39	432
8	Univ Sao Paulo	36	435
9	Univ So Calif	29	125
10	Univ Calif Los Angeles	28	241

### Journal distribution

Based on the retrieved results, papers on VKHD research were distributed among 261 journals. Among them, the journal with the greatest number of articles was *Ocular Immunology and Inflammation*, with 114, followed by the *American Journal of Ophthalmology* and *British Journal of Ophthalmology*. The journal with the highest cited frequency is the *American Journal of Ophthalmology*, followed by *Ophthalmology*, *Ocul Immunol Inflamm*, *Br J Ophthalmol*, and *Invest Ophth Vis Sci*. All of the abovementioned journals are ophthalmology-related journals (Table 4).

**Table 4:** Top 10 source journals

Rank	Journal	Local citations	Counts	H-index
1	AM J Ophthalmol	3097	58	31
2	Ophthalmology	1537	15	15
3	Ocul Immunol Inflamm	1394	114	24
4	Br J Ophthalmol	1254	48	21
5	Invest Ophth Vis Sci	1098	39	20
6	Arch Ophthalmol-Chic	841	13	10
7	Retina-J Ret Vit Dis	774	28	15
8	Jpn J Ophthalmol	767	42	17
9	International Ophthalmology	636	20	9
10	Graef Arch Clin Exp	587	38	15

### Keyword co-occurrence and topic cluster analysis

Among the top 25 keywords, the keywords with the strongest burst were optical coherence tomography, genome-wide association and antigen. The keywords with the longest duration of use were cerebrospinal fluid, antigen, and association. Among these keywords, inflammation, VKH disease, diagnosis, sunset glow fundus, and standardization had become more prevalent in recent years.

**Fig. 5:**
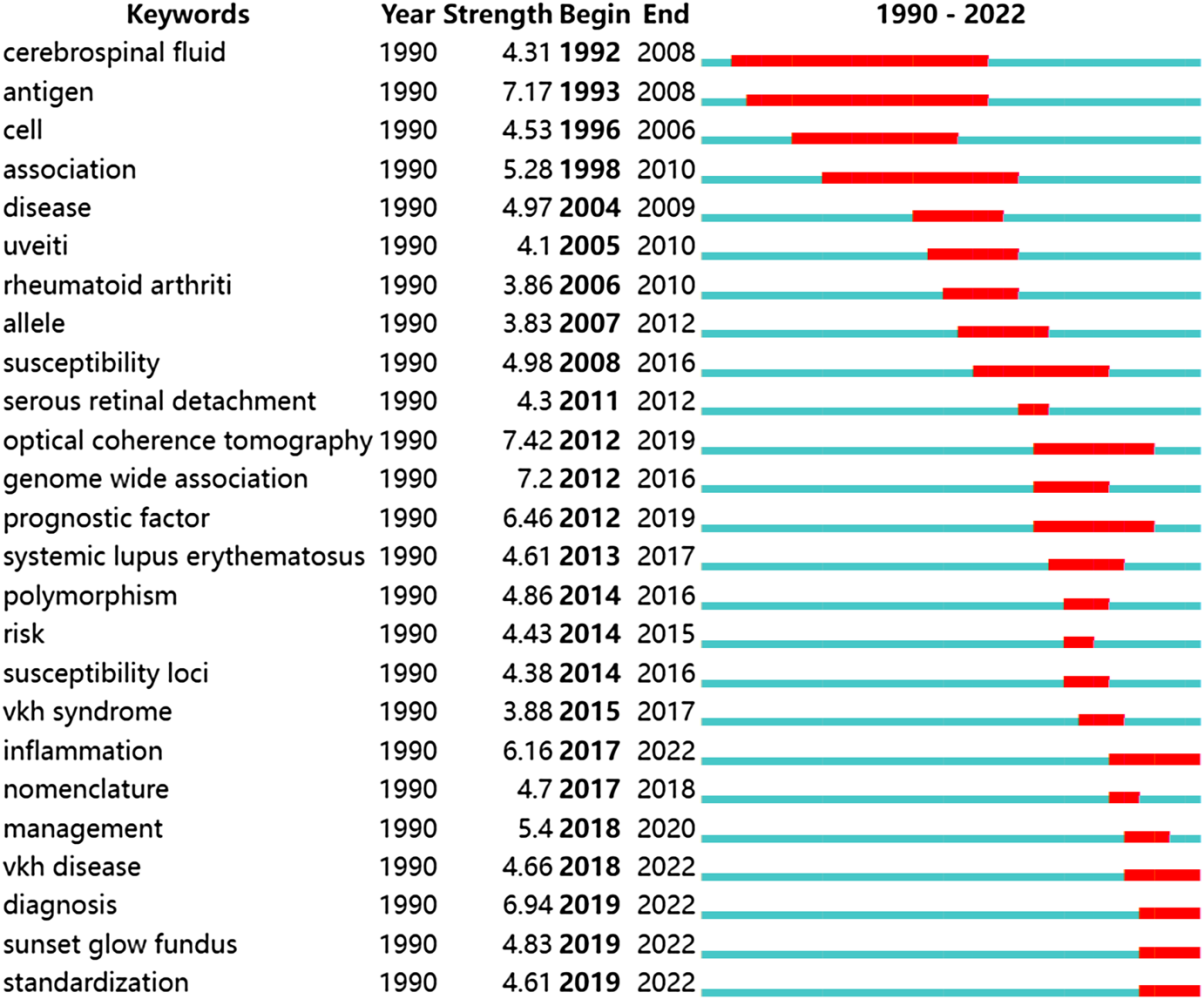
Top 25 keywords with the strongest citation bursts

Apart from “uveitis”, “VKH syndrome”, “VKH disease”, and “disease”, which are parts of the names of VKHD itself, we can mainly divide the remaining keywords into the following two categories. The first category is keywords related to clinical diagnosis, including nomenclature, standardization, diagnosis, optical coherence tomography, sunset glow fundus, polymorphism, inflammation, and serous retinal detachment. The second category is keywords related to pathogenesis research, including antigen, genome-wide association, cerebrospinal fluid, susceptibility, allele, susceptibility loci, and cell (Fig. 5).

## Discussion

VKHD research has had a history of more than 70 years since the disease was officially named. At first, it is divided into Koyanagi disease and Harada disease; the former is mainly caused by recurrent anterior uveitis, while the latter is characterized primarily by exudative retinal detachment and posterior uveitis. However, through many clinical observations, it has been found that these two diseases are different stages of the same disease. As a complex ophthalmic immune disease, the prognosis of VKHD is often polarized. The occurrence of Harada disease usually means that the disease has entered the late stage, resulting in a poor prognosis. Although VKHD can be diagnosed more accurately through certain neurological and skin changes accompanied by VKHD, not all VKHD is typical. For example, VKHD is generally found in adults, but it has also been reported in children [5–10]. Multiple studies have demonstrated better outcomes with early treatment [17, 18]. Therefore, there is a need for scientific diagnostic criteria for the disease.

Since the 1990s, the popularity of optical coherence tomography (OCT) and the use of indocyanine green radiography have greatly helped in the diagnosis of VKHD, and the diagnostic criteria have been continuously improved with the help of innovative examination technology. To date, the aetiology of VKHD has remained unclear, and research on VKHD is still ongoing. This study explores developments and current trends in VKHD research through bibliometric methods.

### Global contributions to VKHD research

From January 1975 to October 2022, we searched a total of 1050 articles related to VKHD involving 261 journals. Japan published the most papers, followed by China and the United States.

According to the number of articles, we can divide the VKHD literature into three stages. The first is from 1975 to 1988. During this stage, the number of papers published was relatively low, with an annual average of 2.6 publications, reflecting the initial lack of understanding of the disease since it was officially named VKHD in 1949. At the beginning of this stage, the American Uveitis Society (AUS) developed a set of diagnostic criteria to draw attention to and help people recognize the disease [19]. Although it has some limitations as the first set of diagnostic criteria for VKHD, it still includes most of its clinical features. The second stage was from 1989 to 2003, during which 170 papers were published, with an average of 11.3 annual publications, a significant increase over the previous stage. In 2001, Read et al. proposed a more complete Revised diagnostic criteria for Vogt‒Koyanagi‒Harada disease based on AUS, which continues to be in use today [7]. In addition, OCT, an essential diagnostic tool for VKHD [20–22], appeared in the 1990s and became of great help in research on VKHD at this stage. In the third stage, spanning 2004 to 2022, 844 articles were published, with an average of 44.4 annual publications. During this period, Japan, China, the Netherlands, Saudi Arabia, and the United States ranked among the top five in the number of publications and contributed significantly to VKHD research. As seen from the figure (Coauthorship-AND-Organizations-30), the cooperation between these five countries is also very close. In addition, at this stage, Yang et al. proposed diagnostic criteria for VKHD based on Chinese data [23]. The increase in the number of articles published in the first two stages was often accompanied by progress in VKHD clinical diagnosis. In contrast, the third stage witnessed the emergence of a substantial amount of basic research literature on molecular biology, which also constitutes the current research trend.

In 2001, the article titled “Revised diagnostic criteria for Vogt‒Koyanagi‒Harada disease: report of an international committee on nomenclature”, the most frequently cited article, was published in the *American Journal of Ophthalmology*. It can also be seen that the criteria proposed by this study have a high degree of recognition and authority. It is more detailed than the AUS criteria and includes two essential examinations: fluorescein angiography and ultrasonography. In addition, the diagnostic criteria allow VKHD to be divided into complete, incomplete, and probable Vogt‒Koyanagi‒Harada disease, which makes them more accurate and consistent with the clinical situation. Although this standard has been widely adopted by peers worldwide [24–26], detailed regulations lead to more examinations, which to some extent increases the costs for patients. We can see the continuous improvement in diagnostic criteria driving VKHD research.

### Focus on VKHD research

According to the cluster analysis, we can divide the literature searched from WoS into three categories. The first category is the essential study to explore the aetiology of VKHD, which yielded the greatest number of studies among the three categories.

The aetiology of VKHD has consistently been a focus of research attention.

As an autoimmune disease, VKHD has been the subject of ongoing immunological research regarding its pathogenesis. As early as 1982, studies found that cytotoxic T lymphocytes may be involved in the occurrence of VKHD [27]; recently, T helper 1 (Th1) cells, Th17 cells, and natural regulatory T cells (Tregs) were also found to play an important role. It is generally accepted that the immune response caused by Th1 and Th17 lymphocytes in patients with VKHD cannot be inhibited by nTreg cells, leading to autoimmune issues. In recent years, the primary purpose of this research has been to explore the influence of molecules upstream and downstream of T cells on the progression of VKHD by regulating T cells [28–38]. Nu. C. et al. found that overexpressed kallistatin can aggravate experimental autoimmune uveitis (EAU) by accelerating the differentiation of Th17 cells [37]. Wang C et al. found a significant decrease in the levels of the immune regulatory molecule progranulin in active VKH patients. When EAU occurred in PGRN^−/−^ mice, Th1 and Th17 cells increased significantly, while Treg cells decreased [36].

Another research direction is to decipher the aetiology of VKHD from the perspective of genetics. In related experiments, whole blood is selected for gene sequencing to screen out susceptible genes. Some studies focused on human leukocyte antigen (HLA)-associated genes and VKH susceptibility, and HLA-DRB1, HLA-DR4/DRw53, and HLA-DR1 alleles are found to be the most susceptible gene phenotypes [39–48]. HLA genes play leading roles in antigen presentation to T lymphocytes, but HLA-associated genes are not the real cause of the disease; they merely serve as genetic markers. Researchers have continuously reviewed the relevant research since 1980, and two related keywords (genome-wide association, susceptibility loci) in the top 25 keywords were related to genetic analysis. The periods over which these terms frequently appeared (2012–2016 and 2014–2016, respectively) overlapped with the year in which the third-generation single-molecule real-time sequencing technology emerged [49]. However, it is worth noting that almost all VKHD is sporadic and does not conform to Mendelian inheritance [1]. With improvements in gene sequencing technology, an increasing number of studies in will emerge this direction.

In addition to the study of genetic susceptibility, virus infection is another important research hotspot [32, 50–55]. Some scholars believe that viral infection may be an important cause of VKHD. The clinical samples involved in such studies include cerebrospinal fluid aqueous humour and serum, and the types of viruses tested include herpes simplex virus (HSV), herpes zoster virus (VZV), cytomegalovirus (CMV), Epstein‒Barr virus (EBV) and human herpes virus type 6 (HHV-6) [32, 50–52]. The research trends related to viral infection have declined in the past five years, but it is worth noting that many clinical studies and case reports in the past three years found that VKHD was associated with COVID-19 as well as with the COVID-19 vaccine [54–61]. However, research on the basis for this hypothesis is still lacking.

The mainstream view is that these three pathogeneses are not isolated, and virus infection often acts as the trigger factor. In addition, patients with susceptibility gene alleles eventually experience attacks to target cells or target tissues under the action of different kinds of immune cells and related effector molecules, resulting in the production of VKHD.

The second cluster of studies was related to auxiliary examination and treatment. As a keyword, optical coherence tomography (OCT) had the highest strength of 7.42, frequently appearing in the related literature from 2012 to 2019. As a standard clinical examination method, different forms of OCT, such as OCT-angiography and enhanced depth imaging (EDI)-OCT, can quickly detect pathological changes in the retina and choroid and consequently be used to monitor the activity of chronic VKHD [20, 22, 62]. As a noninvasive examination with high accuracy and repeatability, OCT has been the subject of many studies for early recognition and evaluation of the prognosis of VKHD in recent years, demonstrating that it has become a popular research topic [63–65]. OCT can effectively detect characteristic serous retinal detachment and macular oedema in VKHD. In the most cited study in this research direction, Fong et al. used EDI-OCT, found that at multiple stages, VKHD can result in permanent changes to small choroidal vessels [66].

ICGA and FFA are commonly used fundus angiography techniques in clinical practice that can detect or monitor the recurrence of VKHD early [67–69]. The difference is that ICGA is more advantageous in choroidal angiography [69, 70]. Therefore, in the study of VKHD, a greater number of articles are written for the ICGA method than for FFA. Other auxiliary examinations include ultrabiomicroscopy [71] and in vivo confocal microscopy [72], but few studies use these kinds of examination alone as a research method.

The number of studies on VKHD treatment is relatively small. Early diagnosis and correct use of adequate systemic corticosteroids can effectively alleviate the symptoms of the disease and reduce the risk of it developing to the chronic recurrent stage [73]. Some immunosuppressive agents, such as methotrexate and cyclosporine, have been used for nearly 30 years [74, 75]. In addition, relatively newer drugs are mainly biologic agents, such as TNF-α inhibitors, infliximab, and interferon alpha-2B [9, 76–80]. However, except for TNF-α inhibitors, very few studies have addressed the other agents. We do not seem to have made much progress in therapeutic methods for treating VKHD. Currently, the main research direction is in-depth basic research on the therapeutic mechanism of known therapeutic drugs [73, 77, 81].

The third cluster of literature is epidemiological investigations and diagnostic criteria. The authors of these studies are in countries or regions with high incidences of VKHD. Retrospective studies conducted by the authors through the case information collected in these regions over the years can often serve as an essential reference for the incidence of VKHD in those regions. As previously mentioned, the diagnostic criteria of VKHD include the American Uveitis Society (AUS) criteria [19], the Revised Diagnostic Criteria for VKH Disease [7], and the Chinese Criteria by Yang PZ [23]. In addition, the STANDARDIZATION OF UVEITIS NOMENCLATURE (SUN) WORKING GROUP has recently developed a new set of classification criteria based on a machine learning method [82]; in this method, VKHD is divided into early and late stages. The main features of the former include the following:

(1) Exudative retinal detachment with a characteristic appearance on fluorescein angiography or optical coherence tomography or (2) panuveitis with ≥ 2 of 5 neurologic symptoms/signs; the main distinguishing point of the latter included a history of early-stage VKH and either (1) sunset glow fundus or (2) uveitis and ≥ 1 of 3 cutaneous signs. However, among these standards, the one with the most far-reaching influence and that was the most frequently cited was the Revised Diagnostic Criteria for VKH Disease developed by Read et al.

## Conclusion

Through bibliometric research, we found that with continuous in-depth mechanistic research and several revisions of diagnostic criteria, researchers have become increasingly aware of and attached importance to VKHD, a complex ophthalmic disease. Currently, one pressing research direction is to understand the relationship between COVID-19 or COVID-19 vaccines and VKHD and the specific mechanisms underlying this relationship. Bibliometric analysis, with its dynamic and visualization tools, provides a comprehensive overview of the research history, emerging trends, and key areas of investigation in VKHD. It serves as a valuable tool for identifying research gaps and areas that require further exploration. Our study has uncovered potential directions for future VKHD research, potentially including delving into the specific molecular mechanisms underlying the disease, exploring the clinical utility of optical coherence tomography angiography and other diagnostic techniques, and conducting clinical studies on novel therapeutic drugs.

### Supplementary Information

Below is the link to the electronic supplementary material.

Supplementary file1 (DOCX 27 kb)

## Data Availability

All data generated or analysed during this study are included in this published article and its supplementary information files.
