# Supplementary material for: Bibliometric analysis of the Vogt‒Koyanagi‒Harada disease literature
Source: Int Ophthalmol. 2023 Aug 8;43(11):4137–50. doi: 10.1007/s10792-023-02815-x (PMC10520158; doi:10.1007/s10792-023-02815-x)
Supplement: Supplementary file 1 — Supplementary file1 (DOCX 27 kb) [file 10792_2023_2815_MOESM1_ESM.docx]

Supplementary table 1: Top 50 most cited articles (Citation counts are based on the retrieval results as of October 28, 2022.)

| Document | Year | Local Citations | Global Citations | DOI |
| --- | --- | --- | --- | --- |
| READ RW, 2001, AM J OPHTHALMOL | 2001 | 504 | 741 | 10.1016/S0002-9394(01)00925-4 |
| MOORTHY RS, 1995, SURV OPHTHALMOL | 1995 | 276 | 436 | 10.1016/S0039-6257(05)80105-5 |
| YANG PZ, 2007, OPHTHALMOLOGY | 2007 | 154 | 192 | 10.1016/j.ophtha.2006.07.040 |
| RUBSAMEN PE, 1991, ARCH OPHTHALMOL-CHIC | 1991 | 106 | 148 | 10.1001/archopht.1991.01080050096037 |
| SNYDER DA, 1980, AM J OPHTHALMOL | 1980 | 101 | 170 | 10.1016/S0002-9394(14)75078-0 |
| SUGIURA S, 1978, JPN J OPHTHALMOL | 1978 | 99 | 140 | 10.1001/jama.1978.03290060031006 |
| YANG PZ, 2005, CURR EYE RES | 2005 | 97 | 220 | 10.1080/02713680500263606 |
| YAMAKI K, 2000, J IMMUNOL | 2000 | 89 | 138 | 10.4049/jimmunol.165.12.7323 |
| CHI W, 2007, J ALLERGY CLIN IMMUN | 2007 | 76 | 159 | 10.1016/j.jaci.2007.01.010 |
| SUGITA S, 2006, INVEST OPHTH VIS SCI | 2006 | 75 | 117 | 10.1167/iovs.05-1547 |
| ISLAM SMM, 1994, INVEST OPHTH VIS SCI | 1994 | 74 | 80 | Not available |
| OHNO S, 1977, AM J OPHTHALMOL | 1977 | 73 | 117 | 10.1016/0002-9394(77)90142-8 |
| RAO NA, 2010, OPHTHALMOLOGY | 2010 | 72 | 99 | 10.1016/j.ophtha.2009.08.030 |
| SHINDO Y, 1994, BRIT J OPHTHALMOL | 1994 | 69 | 100 | 10.1136/bjo.78.3.223 |
| DU LP, 2016, PROG RETIN EYE RES | 2016 | 68 | 102 | 10.1016/j.preteyeres.2016.02.002 |
| BENIZ J, 1991, RETINA-J RET VIT DIS | 1991 | 64 | 97 | 10.1097/00006982-199111030-00001 |
| O'KEEFE GAD, 2017, SURV OPHTHALMOL | 2017 | 62 | 104 | 10.1016/j.survophthal.2016.05.002 |
| GOCHO K, 2001, INVEST OPHTH VIS SCI | 2001 | 61 | 91 | Not available |
| MARUKO I, 2011, RETINA-J RET VIT DIS | 2011 | 56 | 271 | 10.1097/IAE.0b013e3181eef053 |
| ABU EL-ASRAR AM, 2013, ACTA OPHTHALMOL | 2013 | 56 | 76 | 10.1111/aos.12127 |
| NOROSE K, 1996, BRIT J OPHTHALMOL | 1996 | 55 | 74 | 10.1136/bjo.80.11.1002 |
| BOUCHENAKI N, 2001, OPHTHALMOLOGY | 2001 | 55 | 82 | 10.1016/S0161-6420(00)00428-0 |
| LAVEZZO MM, 2016, ORPHANET J RARE DIS | 2016 | 55 | 105 | 10.1186/s13023-016-0412-4 |
| FANG W, 2008, CURR EYE RES | 2008 | 54 | 89 | 10.1080/02713680802233968 |
| READ RW, 2006, AM J OPHTHALMOL | 2006 | 53 | 69 | 10.1016/j.ajo.2006.02.049 |
| NAKAI K, 2012, GRAEF ARCH CLIN EXP | 2012 | 53 | 109 | 10.1007/s00417-011-1910-7 |
| DAMICO FM, 2005, INVEST OPHTH VIS SCI | 2005 | 50 | 73 | 10.1167/iovs.04-1273 |
| KEINO H, 2002, GRAEF ARCH CLIN EXP | 2002 | 49 | 55 | 10.1007/s00417-002-0538-z |
| PERRY HD, 1977, AM J OPHTHALMOL | 1977 | 48 | 104 | 10.1016/0002-9394(77)90623-7 |
| DAVIS JL, 1990, OPHTHALMOLOGY | 1990 | 48 | 98 | 10.1016/s0161-6420(90)32446-6 |
| PAREDES I, 2006, OCUL IMMUNOL INFLAMM | 2006 | 48 | 67 | 10.1080/09273940500536766 |
| GOTO H, 2007, JPN J OPHTHALMOL | 2007 | 48 | 165 | 10.1007/s10384-006-0383-4 |
| OHGURO N, 2012, JPN J OPHTHALMOL | 2012 | 48 | 137 | 10.1007/s10384-012-0158-z |
| NAKAYAMA M, 2012, RETINA-J RET VIT DIS | 2012 | 47 | 135 | 10.1097/IAE.0b013e318256205a |
| FORSTER DJ, 1990, ARCH OPHTHALMOL-CHIC | 1990 | 46 | 70 | 10.1001/archopht.1990.01070120069031 |
| RAO NA, 1997, EYE | 1997 | 45 | 74 | 10.1038/eye.1997.54 |
| SAKAMOTO T, 1991, ARCH OPHTHALMOL-CHIC | 1991 | 44 | 75 | 10.1001/archopht.1991.01080090096030 |
| BYKHOVSKAYA I, 2005, AM J OPHTHALMOL | 2005 | 44 | 64 | 10.1016/j.ajo.2005.04.052 |
| SHINDO Y, 1994, HUM IMMUNOL | 1994 | 43 | 69 | 10.1016/0198-8859(94)90257-7 |
| HOU SP, 2014, NAT GENET | 2014 | 42 | 67 | 10.1038/ng.3061 |
| CHEE SP, 2009, AM J OPHTHALMOL | 2009 | 41 | 59 | 10.1016/j.ajo.2008.07.044 |
| ZHAO M, 1991, ARCH OPHTHALMOL-CHIC | 1991 | 39 | 54 | 10.1001/archopht.1991.01080030070041 |
| GOLDBERG AC, 1998, HUM IMMUNOL | 1998 | 39 | 57 | 10.1016/S0198-8859(97)00265-6 |
| YAMAKI K, 2000, EXP EYE RES | 2000 | 39 | 57 | 10.1006/exer.2000.0893 |
| SUGITA S, 1996, INT IMMUNOL | 1996 | 38 | 72 | 10.1093/intimm/8.5.799 |
| TABBARA KF, 1998, ACTA OPHTHALMOL SCAN | 1998 | 37 | 55 | 10.1034/j.1600-0420.1998.760619.x |
| INOMATA H, 2001, AM J OPHTHALMOL | 2001 | 36 | 52 | 10.1016/S0002-9394(00)00851-5 |
| FONG AHC, 2011, RETINA-J RET VIT DIS | 2011 | 35 | 164 | 10.1097/IAE.0b013e3182083beb |
| CHAN CC, 1988, AM J OPHTHALMOL | 1988 | 34 | 57 | 10.1016/0002-9394(88)90052-9 |
| IMAI YM, 2001, CURR EYE RES | 2001 | 34 | 43 | 10.1076/ceyr.22.4.312.5510 |

Supplementary table 2: Articles with an impact factor (IF) greater than 10 (based on the latest 2022 IF).

| Authors | Title | Journal | IF | DOI |
| --- | --- | --- | --- | --- |
| Hou, SP; Du, LP; Lei, B; Pang, CP; Zhang, MF; Zhuang, WJ; Zhang, ML; Huang, LL; Gong, B; Wang, ML; Zhang, Q; Hu, K; Zhou, QY; Qi, J; Wang, CK; Tian, Y; Ye, Z; Liang, L; Yu, HS; Li, H; Zhou, Y; Cao, QF; Liu, YJ; Bai, L; Liao, D; Kijlstra, A; Xu, JF; Yang, ZL; Yang, PZ | Genome-wide association analysis of Vogt-Koyanagi-Harada syndrome identifies two new susceptibility loci at 1p31.2 and 10q21.3 | NATURE GENETICS | 41.307 | 10.1038/ng.3061 |
| Zhou, QY; Hou, SP; Liang, L; Li, XY; Tan, XY; Wei, L; Lei, B; Kijlstra, A; Yang, PZ | MicroRNA-146a and Ets-1 gene polymorphisms in ocular Behcet's disease and Vogt-Koyanagi-Harada syndrome | ANNALS OF THE RHEUMATIC DISEASES | 27.4 | 10.1136/annrheumdis-2012-201627 |
| Nomura, S; Matsuzaki, T; Ozaki, Y; Yamaoka, M; Yoshimura, C; Katsura, K; Xie, GL; Kagawa, H; Ishida, T; Fukuhara, S | Clinical significance of HLA-DRB1*0410 in Japanese patients with idiopathic thrombocytopenic purpura | BLOOD | 20.3 | Not available |
| Du, LP; Kijlstra, A; Yang, PZ | Vogt-Koyanagi-Harada disease: Novel insights into pathophysiology, diagnosis and treatment | PROGRESS IN RETINAL AND EYE RESEARCH | 17.8 | 10.1016/j.preteyeres.2016.02.002 |
| Li, H; Xie, LH; Zhu, L; Li, ZH; Wang, R; Liu, XX; Huang, ZH; Chen, BY; Gao, YA; Wei, L; He, C; Ju, R; Liu, YZ; Liu, XL; Zheng, YF; Su, WR | Multicellular immune dynamics implicate PIM1 as a potential therapeutic target for uveitis | NATURE COMMUNICATIONS | 16.6 | 10.1038/s41467-022-33502-7 |
| Wang, CK; Zhou, WJ; Su, GN; Hu, JP; Yang, PZ | Progranulin Suppressed Autoimmune Uveitis and Autoimmune Neuroinflammation by Inhibiting Th1/Th17 Cells and Promoting Treg Cells and M2 Macrophages | NEUROLOGY-NEUROIMMUNOLOGY & NEUROINFLAMMATION | 14.2 | 10.1212/NXI.0000000000001133 |
| Chi, W; Yang, PZ; Li, B; Wu, CY; Jin, HL; Zhu, XF; Chen, LN; Zhou, HY; Huang, XK; Kijlstra, A | IL-23 promotes CD4(+) T cells to produce IL-17 in Vogt-Koyanagi-Harada disease | JOURNAL OF ALLERGY AND CLINICAL IMMUNOLOGY | 14.2 | 10.1016/j.jaci.2007.01.010 |
| Yu, HS; Zheng, MM; Zhang, LJ; Li, H; Zhu, YY; Cheng, L; Li, L; Deng, BL; Kijlstra, A; Yang, PZ | Identification of susceptibility SNPs in IL10 and IL23R-IL12RB2 for Behcet's disease in Han Chinese | JOURNAL OF ALLERGY AND CLINICAL IMMUNOLOGY | 14.2 | 10.1016/j.jaci.2016.05.024 |
| Yang, PZ; Liu, SL; Zhong, ZY; Du, LP; Ye, Z; Zhou, WJ; Kijlstra, A | Comparison of Clinical Features and Visual Outcome between Sympathetic Ophthalmia and Vogt-Koyanagi-Harada Disease in Chinese Patients | OPHTHALMOLOGY | 13.7 | 10.1016/j.ophtha.2019.03.049 |
| Rao, NA; Gupta, A; Dustin, L; Chee, SP; Okada, AA; Khairallah, M; Bodaghi, B; Lehoang, P; Accorinti, M; Mochizuki, M; Prabriputaloong, T; Read, RW | Frequency of Distinguishing Clinical Features in Vogt-Koyanagi-Harada Disease | OPHTHALMOLOGY | 13.7 | 10.1016/j.ophtha.2009.08.030 |
| Hou, SP; Liao, D; Zhang, J; Fang, J; Chen, L; Qi, J; Zhang, Q; Liu, YJ; Bai, L; Zhou, Y; Kijlstra, AZ; Yang, PZ | Genetic Variations of IL17F and IL23A Show Associations with Behcet's Disease and Vogt-Koyanagi-Harada Syndrome | OPHTHALMOLOGY | 13.7 | 10.1016/j.ophtha.2014.09.025 |
| Rathinam, SR; Namperumalsamy, P; Nozik, RA; Cunningham, ET | Vogt-Koyanagi-Harada syndrome after cutaneous injury | OPHTHALMOLOGY | 13.7 | 10.1016/S0161-6420(99)90129-X |
| Yang, PZ; Ren, YL; Li, B; Fang, W; Meng, QL; Kijlstra, A | Clinical characteristics of Vogt-Koyanagi-Harada syndrome in Chinese patients | OPHTHALMOLOGY | 13.7 | 10.1016/j.ophtha.2006.07.040 |
| Bouchenaki, N; Herbort, CP | The contribution of indocyanine green angiography to the appraisal and management of Vogt-Koyanagi-Harada disease | OPHTHALMOLOGY | 13.7 | 10.1016/S0161-6420(00)00428-0 |
| Yeh, S; Karne, NK; Kerkar, SP; Heller, CK; Palmer, DC; Johnson, LA; Li, ZQ; Bishop, RJ; Wong, WT; Sherry, RM; Yang, JC; Dudley, ME; Restifo, NP; Rosenberg, SA; Nussenblatt, RB | Ocular and Systemic Autoimmunity after Successful Tumor-Infiltrating Lymphocyte Immunotherapy for Recurrent, Metastatic Melanoma | OPHTHALMOLOGY | 13.7 | 10.1016/j.ophtha.2008.12.004 |
| Chang, R; Chen, L; Su, GN; Du, LP; Qin, Y; Xu, J; Tan, HD; Zhou, CJ; Cao, QF; Yuan, GX; Kijlstra, A; Yang, PZ | Identification of Ribosomal Protein S4, Y-Linked 1 as a cyclosporin A plus corticosteroid resistance gene | JOURNAL OF AUTOIMMUNITY | 13.7 | 10.1016/j.jaut.2020.102465 |
| Anquetil, C; Salem, JE; Lebrun-Vignes, B; Touhami, S; Desbois, AC; Maalouf, G; Domont, F; Allenbach, Y; Cacoub, P; Bodaghi, B; Saadoun, D | Evolving spectrum of drug-induced uveitis at the era of immune checkpoint inhibitors results from the WHO's pharmacovigilance database | JOURNAL OF AUTOIMMUNITY | 13.7 | 10.1016/j.jaut.2020.102454 |
| Ganesh, SK; Padmaja, MS; Babu, K; Biswas, J | Cataract surgery in patients with Vogt-Koyanagi-Harada syndrome | JOURNAL OF CATARACT AND REFRACTIVE SURGERY | 13.7 | 10.1016/S0886-3350(03)00552-2 |
| Silpa-archa, S; Silpa-archa, N; Preble, JM; Foster, CS | Vogt-Koyanagi-Harada syndrome: Perspectives for immunogenetics, multimodal imaging, and therapeutic options | AUTOIMMUNITY REVIEWS | 13.6 | 10.1016/j.autrev.2016.04.001 |
| Sakata, VM; da Silva, FT; Hirata, CE; de Carvalho, JF; Yamamoto, JH | Diagnosis and classification of Vogt-Koyanagi-Harada disease | AUTOIMMUNITY REVIEWS | 13.6 | 10.1016/j.autrev.2014.01.023 |
| Greco, A; Fusconi, M; Gallo, A; Turchetta, R; Marinelli, C; Macri, GF; De Virgilio, A; de Vincentiis, M | Vogt-Koyanagi-Harada syndrome | AUTOIMMUNITY REVIEWS | 13.6 | 10.1016/j.autrev.2013.01.004 |
| Hu, YJ; Hu, YX; Xiao, YH; Wen, F; Zhang, SC; Liang, D; Su, LS; Deng, Y; Luo, JW; Ou, JS; Lu, MZ; Hong, YH; Chi, W | Genetic landscape and autoimmunity of monocytes in developing Vogt-Koyanagi-Harada disease | PROCEEDINGS OF THE NATIONAL ACADEMY OF SCIENCES OF THE UNITED STATES OF AMERICA | 11.1 | 10.1073/pnas.2002476117 |
